# Carfilzomib potentiates CUDC-101-induced apoptosis in anaplastic thyroid cancer

**DOI:** 10.18632/oncotarget.7760

**Published:** 2016-02-26

**Authors:** Lisa Zhang, Myriem Boufraqech, Ross Lake, Electron Kebebew

**Affiliations:** ^1^ Endocrine Oncology Branch, National Cancer Institute, National Institutes of Health, Bethesda, Maryland, USA; ^2^ Laboratory of Genitourinary Cancer Pathogenesis, National Cancer Institute, Bethesda, Maryland, USA

**Keywords:** carfilzomib, CUDC-101, anaplastic thyroid cancer, combination therapy, apoptosis

## Abstract

Anaplastic thyroid cancer (ATC) is one of the most aggressive human malignancies, with no effective treatment currently available. Previously, we identified agents active against ATC cells, both *in vitro* and *in vivo*, using quantitative high-throughput screening of 3282 clinically approved drugs and small molecules. Here, we report that combining two of these active agents, carfilzomib, a second-generation proteasome inhibitor, and CUDC-101, a histone deacetylase and multi-kinase inhibitor, results in increased, synergistic activity in ATC cells. The combination of carfilzomib and CUDC-101 synergistically inhibited cellular proliferation and caused cell death in multiple ATC cell lines harboring various driver mutations observed in human ATC tumors. This increased anti-ATC effect was associated with a synergistically enhanced G2/M cell cycle arrest and increased caspase 3/7 activity induced by the drug combination. Mechanistically, treatment with carfilzomib and CUDC-101 increased p21 expression and poly (ADP-ribose) polymerase protein cleavage. Our results suggest that combining carfilzomib and CUDC-101 would offer an effective therapeutic strategy to treat ATC.

## INTRODUCTION

Anaplastic thyroid cancer (ATC) is a rare, but very aggressive, human malignancy. The approximate incidence of ATC is one to two cases per million per year, but the median survival of ATC patients is only about five months [1-3]. Current treatment regimens fail to provide durable clinical benefits, and patient survival has not been improved in over six decades [1, 2, 4]. Thus, there is an urgent need to develop new, effective treatments to improve ATC patient survival.

To identify therapeutic targets for this lethal disease, extensive genomic and genetic studies have been performed. ATC tumors frequently have mutations in *TP53, BRAF, RAS, β-catenin, PIK3CA,* and *PTEN* [5-11]. Overexpression of epidermal growth factor receptor (EGFR), histone deacetylases (HDACs), β-catenin, aurora kinases, cyclins, platelet-derived growth factor receptor beta (PDGFRB), survivin, and HER-2 are also common [12-20]. Moreover, both the RAS/RAF/MEK/ERK and PI3K/AKT/mTOR pathways are activated in ATC [21, 22]. These findings suggest that there is a high degree of genetic abnormality and substantial aberrant expression of numerous molecules in ATC, leading to dysregulation of multiple signaling pathways [11]. Therefore, targeted therapies, which interfere with only one or a few specific molecule(s), may not offer effective ATC treatment.

Recently, using quantitative high-throughput screening (qHTS) on 3,282 clinically approved drugs and small molecules, we identified several agents that are active in ATC cells, both *in vitro* and *in vivo* [23-26]. Among these were carfilzomib, a second-generation proteasome inhibitor, and CUDC-101, an inhibitor of HDACs and multiple kinases. It has been reported that, in colorectal cancer cells, the combination of HDACs and proteasome inhibitors results in superior antitumor activity, accompanied by the altered gene expressions associated with cell cycle arrest and apoptosis [27]. Since ATC tumors have a high degree of genomic and genetic abnormality, we hypothesized that targeting multiple altered pathways simultaneously may improve therapeutic efficacy and reduce the effective concentration needed for each drug, thus lessening their potential toxicities. Therefore, we tested the combination of carfilzomib and CUDC-101 as a potential therapeutic strategy in ATC cells. We found that by concurrently inhibiting the proteasome, HDACs, EGFR, and HER2 pathways, the combination of carfilzomib and CUDC-101 synergistically inhibited tumor cell proliferation in multiple ATC cell lines with driver mutations observed in human ATC tumors. The superior anti-ATC activity of the drug combination was associated with synergistically enhanced G2/M cell cycle arrest and caspase-dependent apoptosis. Mechanistically, treatment with carfilzomib and CUDC-101 induced increased p21 expression and augmented poly (ADP-ribose) polymerase (PARP) protein cleavage. Collectively, our results support the combination of carfilzomib and CUDC-101 as a promising ATC treatment strategy.

## RESULTS

### Carfilzomib potentiates the anti-ATC activity of CUDC-101

**Figure 1:**
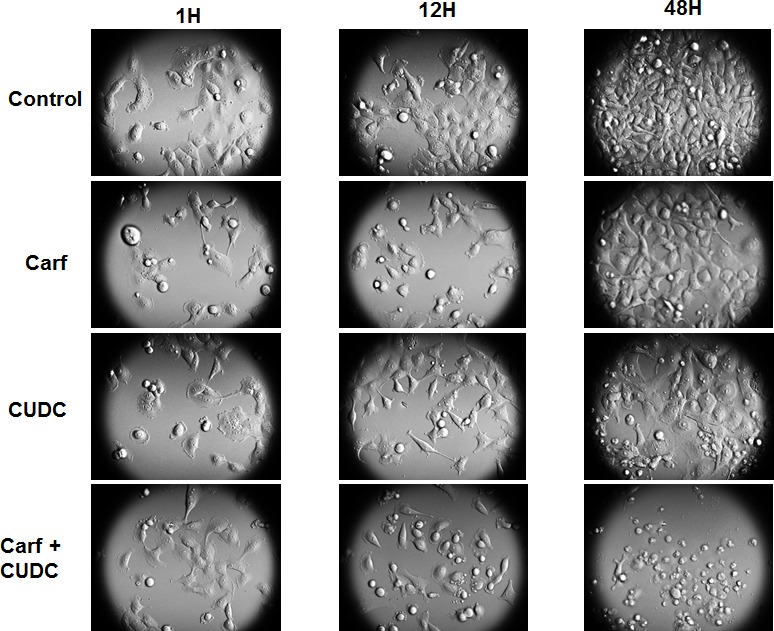
Carfilzomib potentiates CUDC-101-induced anti-ATC effects in 8505c cells Images were captured using a bright-field, time-lapse microscopy imaging system at the indicated times after adding vehicle control, carfilzomib (6 nM), CUDC-101 (0.8 μM), or carfilzomib (6 nM) and CUDC-101 (0.8 μM).

To examine the effects of carfilzomib and CUDC-101 in combination on ATC cell proliferation, time-lapse video microscopy was used to continuously monitor 8505c cell growth in vehicle control, carfilzomib and CUDC-101 individually, and carfilzomib and CUDC-101 combined groups over a 48 h time span. Figure 1 shows the screen capture images from 1-, 12-, and 48 h-treatments. Compared to vehicle control cells, carfilzomib at 6 nM inhibited 8505c cell growth, and induced cell death after 48 h of treatment. At a 0.8 μM concentration, CUDC-101decreased 8505c cell proliferation, and induced cell death after 48 h of treatment. Remarkably, when the same concentrations of carfilzomib and CUDC-101 were combined, cancer cell death increased to a level greater than those attributed to the individual drugs. After 48-hour incubation, most cells in the group treated with carfilzomib and CUDC-101 were dead, with cellular debris evident. Time-lapse microscopy recorded over 48 h showed similar cell viability changes (Supplemental Data).

**Figure 2:**
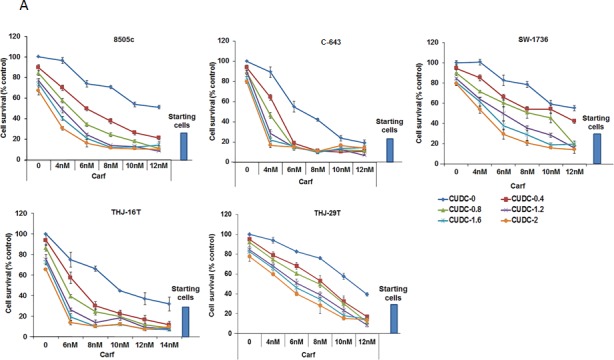

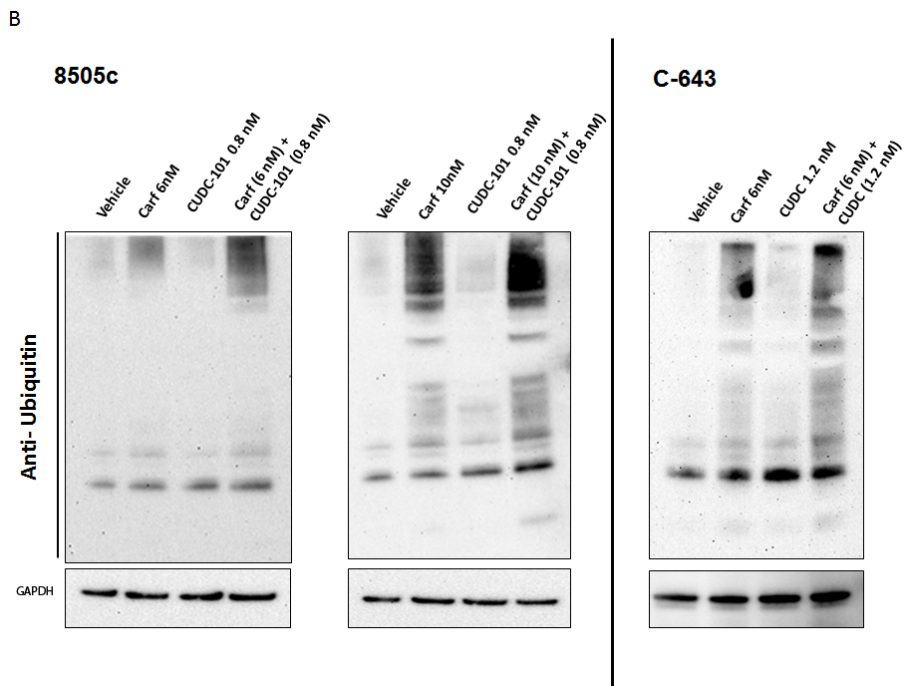
Carfilzomib and CUDC-101 synergistically inhibit cellular proliferation in multiple ATC cell lines **A.** Five different ATC cell lines were tested. Each cell line was treated with five different concentrations of carfilzomib, five different concentrations of CUDC-101, and 25 different combinations of carfilzomib and CUDC-101 at various concentrations, as indicated. Cell proliferation levels were determined after 48-h treatments. The numbers of the vehicle control-treated cells were set as the 100% levels. **B.** Western blot analysis of ubiquitinated proteins after 24h of exposure to carfilzomib treatment (6 and 10 nM for 8505c and 6 nM for C-643), CUDC-101 (0.8 nM for 8505c and 1.2 nM for C-643) or the combination of carfilzomib and CUC-101.

Molecular heterogeneity within tumors is one of the major reasons that anticancer drugs are restricted in their efficacy. To investigate whether the combination of carfilzomib and CUDC-101 would be effective in ATC cells that harbor different driver mutations, we tested the drug combination using five different ATC cell lines, each with distinct genetic backgrounds. 8505c cells have *BRAF V600E, EGFR,* and *TP53* mutations; C-643 cells have *HRAS*, *TP53*, and *PTEN* mutations; SW-1736 cells have *BRAF V600E*, *TP53*, and *PIK3CB* mutations; THJ-16T cells have *TP53*, *RB*, and *PI3KCA* mutations; and THJ-29T cells have an *RB* mutation [23, 28]. The mutations present in these cell lines are frequently observed in ATC tumors, suggesting that they are a good representation of human ATC. In all cell lines tested, the addition of carfilzomib increased CUDC-101 cell proliferation inhibition, and the effect was observed across all carfilzomib concentrations tested (Figure 2A). Furthermore, we confirmed the proteasome inhibitor effect of carfilzomib by measuring the accumulation of ubiquitinated proteins after treatment (Figure 2B). The accumulation of ubiquitinated protein was higher with carfilzomib and CUDC-101 treatment than carfilzomib treatment alone (Figure 2B).

To examine whether the carfilzomib and CUDC-101combination is additive or synergistic, we used the approach of Chou and Talalay to calculate the combination index (CI), in which CI = 1 indicates an additive effect, CI < 1 a synergistic effect, and CI > 1 an antagonistic effect [29]. As shown in Table 1, for all five ATC cell lines, the calculated CIs were less than one for all tested concentrations, indicating a synergistic effect of carfilzomib and CUDC-101, in combination, on cellular proliferation inhibition.

**Table 1:** Combination index (CI) of carfilzomib and CUDC-101 combinations

Cell lines	Carf (nM)	CUDC (μM)	CIÙ
8505c	4.0	0.4	0.71
	6.0	0.8	0.51
	8.0	1.2	0.41
	10.0	1.6	0.48
	12.0	2.0	0.54
C-643	4.0	0.4	0.74
	6.0	0.8	0.48
	8.0	1.2	0.58
	10.0	1.6	0.80
	12.0	2.0	0.97
SW-1736	4.0	0.4	0.81
	6.0	0.8	0.79
	8.0	1.2	0.82
	10.0	1.6	0.86
	12.0	2.0	0.97
THJ-16T	6.0	0.4	0.85
	8.0	0.8	0.61
	10.0	1.2	0.66
	12.0	1.6	0.48
	14.0	2.0	0.68
THJ-29T	4.0	0.4	0.80
	6.0	0.8	0.82
	8.0	1.2	0.74
	10.0	1.6	0.59
	12.0	2.0	0.62

Ù The combination index (CI) was calculated according to the approach described by Chou and Talalay. CI = 1 indicates an additive effect, CI < 1 a synergistic effect, and CI > 1 an antagonistic effect.

### Carfilzomib increases CUDC-101-induced cell cycle arrest and apoptosis in ATC cells

**Figure 3:**
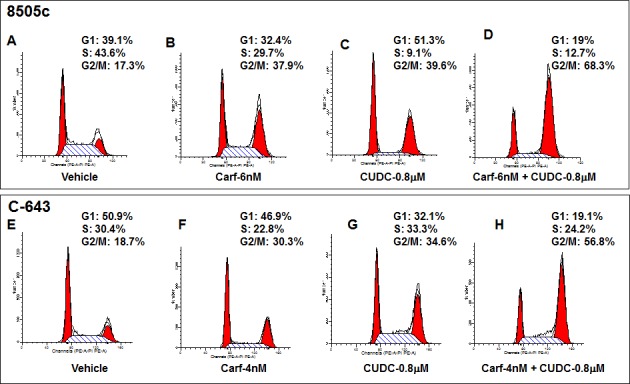
Carfilzomib potentiates the effects of CUDC-101 on cell cycle arrest in 8505c (A to D) and C-643 (E to H) cells Representative FACS data show that the combination of carfilzomib and CUDC-101 increase G2/M cell cycle arrest.

We next examined the underlying mechanism for the increased ATC cell death induced by the combination of carfilzomib and CUDC-101. To study this, we tested two representative ATC cell lines, 8505c and C-643. We first assessed cell cycle progression in the cells when treated with the different agents individually. As we reported previously, either carfilzomib or CUDC-101 alone induced cell cycle arrest in G2/M phase (Figure 3) [23, 24]. In 8505c control cells, 17.3% of the cells were in G2/M phase, and treatment with carfilzomib or CUDC-101 increased the percentage to 37.9% and 39.6%, respectively. Similarly, in C-643 cells, 18.7% of the cells in the vehicle control group were in G2/M phase, and treatment with carfilzomib or CUDC-101 increased the percentage to 30.3% and 34.6%, respectively. Remarkably, the addition of carfilzomib to CUDC-101, in both 8505c and C-643 cells, resulted in a more than 100% increase of cells in G2/M phase when compared to the individual carfilzomib or CUDC-101 treatment groups, suggesting that the drugs synergistically inhibited cell cycle progression in ATC cells.

**Figure 4:**
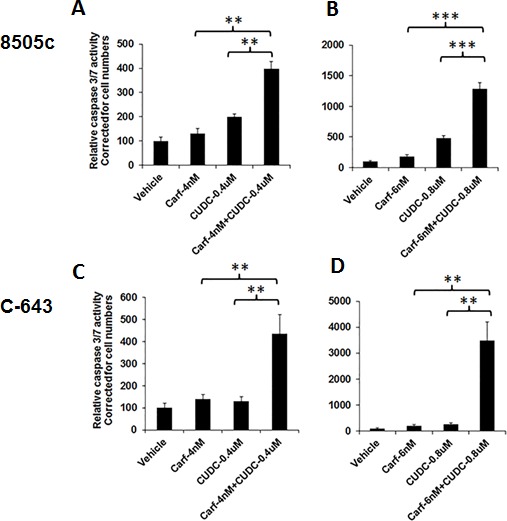
The combination of carfilzomib and CUDC-101 increased caspase-dependent apoptosis 8505c and C-543 cells were treated with drugs at the indicated concentration(s). After 48 h of treatment, the Caspase-Glo 3/7 assay was performed. **P < 0.01, ***P < 0.001.

Caspases play a critical role in inducing apoptosis to cause cell death. To test whether the combination of carfilzomib and CUDC-101 had a synergistic effect on caspase-dependent apoptosis, we performed caspase 3/7 activity assays. In 8505c cells, 4-nM carfilzomib or 0.4-μM CUDC-101 treatments resulted in a 31% and 100% increases in caspase 3/7 activity, respectively. Adding carfilzomib to CUDC-101 led to an almost 300% increase in caspase activity (Figure 4). Similar synergistic effects were also observed at higher drug concentrations, as well as in C-643 cells (Figure 4).

### Combination of carfilzomib and CUDC-101 acts synergistically to increase p21 expression and cleaved PARP levels in ATC cells

**Figure 5:**
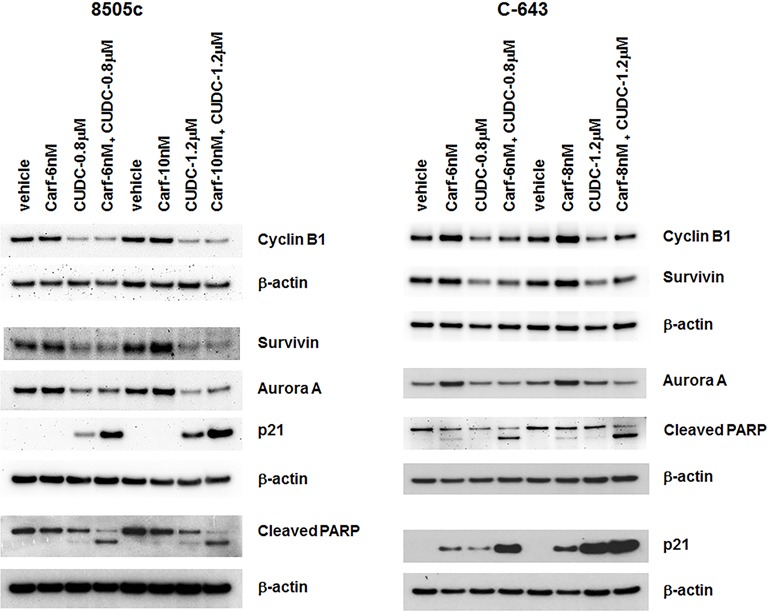
Carfilzomib and CUDC-101 combined synergistically induced p21 expression and increased PARP cleavage in ATC cells 8505c and C-643 cells were treated with different drugs at the indicated concentrations for 24 h. Total cell lysates were analyzed for the indicated proteins. β-actin was used as a loading control.

To understand the mechanism behind how carfilzomib and CUDC-101 synergistically regulate ATC cell proliferation, cell cycle progression, and apoptosis, we examined several key signaling molecules involved in these cellular functions. Cyclin B1 is an important regulatory protein involved in mitosis. Carfilzomib treatment had no significant effect on cyclin B1 expression in 8505c cells, but did result in increased cyclin B1expression in C-643 cells. In contrast, CUDC-101 treatment decreased cyclin B1 expression in both 8505c and C-643 cells. The effects of therapy with carfilzomib and CUDC-101 combined on cyclin B1 expression were similar to those of CUDC-101 treatment alone, suggesting that cyclin B1 did not play an important role in the synergistic effect induced by the drug combination (Figure 5). Similar results were also observed for aurora kinase A and survivin, excluding these proteins from any important role in the drugs’ synergistic functions (Figure 5).

P21 is an important cell cycle regulator. Therefore, we examined the effect(s) of carfilzomib and CUDC-101 individually, as well as in combination, on p21 expression. P21 protein was undetectable in vehicle control-treated 8505c and C-643 cell lines, which matches the aggressive cellular phenotype of ATC. Carfilzomib treatment had no effect on p21 expression in 8505c cells, but it did induce the expression of p21 in C-643 cells, which was consistent with the higher sensitivity of C-643 cells to carfilzomib than that of 8505c cells. CUDC-101 treatment, as previously observed, increased p21 levels in both 8505c and C-643 cells. Carfilzomib significantly potentiated the CUDC-101-induced p21 expression, resulting in the dramatic overexpression of p21 protein (Figure 5).

PARP plays a pivotal role in the detection and repair of DNA damage, and is critical for cancer cell survival and growth when cells are under genotoxic stress. PARP is the primary cleavage target of caspase-3, and serves as a marker of cellular apoptosis. As shown in Figure 5, when used as a single agent, either carfilzomib or CUDC-101 was able to induce a slight increase in PARP cleavage. Adding carfilzomib to CUDC-101 resulted in dramatically enhanced cleavage of PARP in both 8505c and C-643 cells, which explains the synergistic effect of the drug combination on cancer cell apoptosis that we observed.

## DISCUSSION

With our improved understanding of the driver genetic changes involved in cancer initiation and progression, researchers have developed many targeted anticancer therapies. However, the development of drug resistance is common, and many targeted agents show efficacy in only a limited patient population. Lethal cancers, such as ATC, contain multiple mutated genes and dysregulated pathways. Therefore, these tumors are or become resistant to many targeted treatments by bypassing the level and/or factor inhibited by a specific inhibitor [30]. To achieve clinical efficacy, simultaneously inhibiting multiple cancer cell pathways may offer a better treatment strategy. In this study, we evaluated the potential of carfilzomib and CUDC-101 in combination for the treatment of ATC. As previously reported, carfilzomib or CUDC-101 alone displayed an antiproliferative effect in ATC, while the combination of the two drugs profoundly augmented this effect. Our results demonstrated that combining carfilzomib with CUDC-101 synergistically inhibited ATC cell proliferation, enhanced G2/M cell cycle arrest, and increased cancer cell apoptosis. Mechanistically, combining carfilzomib and CUDC-101 resulted in increased p21 expression and PARP cleavage, demonstrating the molecular basis for the enhanced synergistic effect of the drug combination in ATC therapy.

The ubiquitin proteasome pathway is involved in cancer initiation and progression, and angiogenesis in thyroid cancer [31]. Proteasome inhibition has emerged as a new therapeutic option for cancer treatment [32]. Bortezomib, a first generation proteasome inhibitor, has been tested for the treatment of both hematological and solid cancers, including ATC [33, 34]. However, drug resistance and neurotoxicity associated with bortezomib treatment has restricted its clinical efficacy [35]. Carfilzomib is an irreversible inhibitor of the 20S proteasome. Compared to bortezomib, carfilzomib induces a more sustained inhibition of cellular proteasome activity [36]. We have recently shown that carfilzomib is an effective anticancer agent in ATC. Carfilzomib treatment significantly inhibited ATC cell proliferation, and resulted in G2/M cell cycle arrest and caspase-dependent apoptosis. Carfilzomib treatment in mice with established, widely metastatic disease significantly increased their survival [24].

CUDC-101 is a dual inhibitor of EGFR, HER2, and HDACs, and displays potent antiproliferative and proapoptotic activities against cancer cells [37, 38]. A Phase I study of CUDC-101 reported that the drug was tolerable and showed some preliminary evidence of antitumor activity [39]. Through qHTS of 3282 clinically approved drugs and drug candidates in ATC cell lines, we recently demonstrated that CUDC-101 is one of the top active agents against ATC [23]. CUDC-101 inhibited ATC cell proliferation, and resulted in cancer cell death by inducing cell cycle arrest and caspase-dependent apoptosis. Furthermore, using an *in vivo* metastatic ATC mouse model, we found that CUDC-101 treatment inhibited tumor growth and metastases, and significantly prolonged animal survival [23].

Like other aggressive cancers, ATC tumors frequently contain multiple genetic mutations, including *TP53, BRAF, RAS, β-catenin, PIK3CA,* and *PTEN* [5-11]. The overexpression of EGFR, HDACs, β-catenin, aurora kinases, cyclins, PDGFRB, survivin, and HER-2 are also common in ATC [12-20]. The high degree of genetic abnormality and the substantial dysregulated expression of tumor suppressive or growth promoting genes observed in ATC may explain why it has been such a challenge to develop an effective therapeutic strategy for patients with ATC when only a single or a few of these dysregulated genes/pathways are targeted. Indeed, a Phase II trial of pazopanib, a potent multi-targeted receptor tyrosine kinase inhibitor, reported that in patients with ATC, the drug induced only transient disease regression in some of the patients, without any confirmed partial or complete responses [40]. In combination with paclitaxel, efatutazone, an oral PPAR-γ agonist, has been reported to induce a partial response in one out of 15 patients in a Phase I trial in patients with ATC [41]. Similarly, the combination of carboplatin and paclitaxel (CP) and fosbretabulin, a vascular-disrupting agent, did not show a significant difference in progression-free survival between CP and CP/fosbretabulin-treated patients with ATC [42]. Therefore, simultaneous inhibition of multiple pathways that are dysregulated in ATC may be necessary to achieve effective therapies for ATC.

Combinations of proteasome inhibitors and other targeted agents have been tested for use in cancer therapy. In imatinib-sensitive and -resistant chronic myeloid leukemia models, carfilzomib showed a synergistic effect in combination with tyrosine kinase inhibitors [43]. In acute myeloid leukemia cells, proteasome inhibitors could reverse the quizartinib resistance induced by FLT3 kinase domain mutations, suggesting that these compounds may prevent the emergence of mutant clones arising from tyrosine kinase inhibitor treatments [44]. In non-small cell lung cancer cell lines, carfilzomib combined with HDAC inhibitor SAHA demonstrated synergistic anticancer activity [45]. In the current study, we found that the combination of carfilzomib and CUDC-101 induced synergistic anti-ATC activity. The drug combination increased p21 expression and PARP cleavage, and resulted in increased cell cycle arrest and cancer cell apoptosis. Although we did not test the drug combination in an *in vivo* model of ATC, we have previously reported that carfilzomib and CUDC-101 treatment results in potent anticancer activity *in vivo*. In the current study, our focus was to evaluate the effects of the drug combination *in vitro* and to determine the mechanism of the synergistic effects.

In conclusion, we demonstrate that by targeting the HDACs, proteasomes, and EGFR downstream signaling pathways, the combination of carfilzomib and CUDC-101 offers an effective treatment strategy that synergistically inhibits ATC cell proliferation and causes ATC cell death. Since the combination is effective in multiple ATC cell lines that contain major genetic mutations observed in ATC tumors, the present findings provide a rationale to investigate the combination of carfilzomib and CUDC-101 as a potential therapy for ATC.

## MATERIALS AND METHODS

### Cell lines

Human ATC cell line 8505c was purchased from the European Collection of Cell Cultures (Salisbury, United Kingdom); C-643 and SW-1736 were obtained from Cell Lines Service (GmbH, Eppelheim, Germany); and THJ-16T and THJ-29T were kindly provided by Dr. John A. Copland (Department of Cancer Biology, Mayo Clinic, Jacksonville, FL) and have been described previously [23]. All cell lines were authenticated using short tandem repeat profiling. 8505c cells have *BRAF V600E, EGFR,* and *TP53* mutations; C-643 cells harbor *HRAS*, *TP53*, and *PTEN* mutations; SW-1736 has *BRAF V600E*, *TP53*, and *PIK3CB* mutations; THJ-16T has *TP53*, *RB*, and *PI3KCA* mutations; and THJ-29T has an *RB* mutation [28]. Cells were maintained in Dulbecco's modified Eagle's medium (DMEM) supplemented with 10% fetal calf serum (FCS), penicillin (100 U/ml), streptomycin (100 μg/ml), Fungizone (250 ng/ml), TSH (10 IU/l), and insulin (10 μg/ml) in a 5% CO_2_ atmosphere at 37°C.

### Cell proliferation assay

Cell proliferation assays were performed in 96-well plates in quadruplicate. Cells were plated in 96-well black plates at 2×10^3^ cells/well in 100 μl of culture medium. After 24 h (day 0), 100 μl of fresh culture medium containing double concentrations of the indicated drug(s) or vehicle control were added to each well. CyQUANT (Invitrogen) proliferation assays were performed according to the manufacturer's instructions. The cell numbers in the 96-well black plates were determined using a 96-well fluorescence microplate reader (Molecular Devices, Sunnyvale, CA) at 485 nm/538 nm.

### Cell cycle assay

Cells were plated in T25 flasks at a density of 4×10^5^ (8505c) or 3×10^5^ (C-643) cells/flask in 4 ml of culture medium. After 24 h, 4 ml of fresh culture medium containing double concentrations of the indicated drug(s) or vehicle control were added to each flask. Following a 24-h treatment, the cells were harvested, washed and resuspended in PBS, and fixed with ice-cold 70% ethanol at 4°C. After washing with PBS, ribonuclease A and propidium iodide (PI) were added to the cell suspension, which was then incubated at 37°C for 20 min in the dark. A total of 20 000 nuclei were examined by flow cytometry using a CANTO II flow cytometer (Becton Dickinson, Franklin Lakes, NJ, USA). Doublets, cell clumps, and debris were excluded using PI fluorescence pulse width and pulse area measurements. Cell cycle analysis on the gated PI distribution was performed using Modfit software (Verity Software House, Inc., Topsham, ME, USA).

### Apoptosis assay

To determine whether drug treatment resulted in apoptosis, we used the Caspase-Glo 3/7 assay (Promega) to measure caspase activity. 8505c and C-643 cells were plated in white 96-well plates at a density of 2×10^3^ cells/well in 100 μl of culture medium. After 24 h (day 0), 100 μl of fresh culture medium containing double concentrations of the indicated drug(s) or vehicle control were added to each well. After 48 h, cells were analyzed for caspase 3/7 activity using the Caspase-Glo 3/7 assay kit according to the manufacturer's instructions. The relative luminescence (which is proportional to caspase 3/7 activity) was calculated and normalized to the total cell number.

### Western blot and antibodies

Total cell lysates were prepared with 1% SDS and 10 mM Tris buffer (pH 7.4), and separated by SDS-PAGE. After transfer to a nitrocellulose membrane, the proteins were immunoblotted with different antibodies overnight at 4°C. The following antibodies were used: anti-ubiquitin (catalog # 3933) (1:1000), anti-p21 (catalog #: 2947) (1:500), anti-survivin (catalog #: 2808) (1:2000), anti-cleaved PARP (catalog #: 9546) (1:300), and anti-cyclin B1 (catalog #: 12231) (1:1000), all from Cell Signaling Technology (Boston, MA); anti-aurora A from Abcam (Cambridge, MA) (catalog #: ab190367) ; and anti-β-actin (catalog #: sc-81178) (1:3000) from Santa Cruz Biotechnology (Dallas, TX). Anti-human β-actin was used as a loading control. The membranes were incubated with the appropriate HRP-conjugated IgG (anti-rabbit antibody at 1:3000 dilution, Cell Signaling Technology, Danvers, MA, USA, or anti-mouse antibody at 1:10 000 dilution, Santa Cruz Biotechnology). An ECL assay (Thermo Scientific, Rockford, IL, USA) was used to detect the proteins.

### Time-lapse video microscopy

8505C cells were plated in 35-mm coverslip, glass-bottomed culture dishes (MatTek, Ashland, MA) at a density of 2.5×10^4^ cells/dish in 2 ml of culture medium. After 24 h (day 0), 1 ml of culture medium was removed from the dish, and 1 ml of fresh culture medium containing double concentrations of the indicated drug(s) or vehicle control were added. The plates were then loaded into Olympus VivaView incubator (Center Valley, PA), and bright-field time-lapse images of cells were taken in a temperature- and humidity-controlled environment every 10 min for 48 h using a UPLSAPO40X objective with a 0.5X magnification changer.

### Statistical analyses

Two-sided *t*-tests were used to assess differences in the Caspase-Glo 3/7 and cell proliferation assay results. A *p* value < 0.05 was considered statistically significant. GraphPad Prism 6 (La Jolla, CA) and CompuSyn (ComboSyn, Inc, New York, NY) were used for statistical analysis.

## SUPPLEMENTARY FIGURES AND TABLES









## References

[1] Smallridge RC, Ain KB, Asa SL, Bible KC, Brierley JD, Burman KD, Kebebew E, Lee NY, Nikiforov YE, Rosenthal MS, Shah MH, Shaha AR, Tuttle RM, American Thyroid Association Anaplastic Thyroid Cancer Guidelines T (2012). American Thyroid Association guidelines for management of patients with anaplastic thyroid cancer. Thyroid.

[2] Kebebew E, Greenspan FS, Clark OH, Woeber KA, McMillan A (2005). Anaplastic thyroid carcinoma. Treatment outcome and prognostic factors. Cancer.

[3] Nagaiah G, Hossain A, Mooney CJ, Parmentier J, Remick SC (2011). Anaplastic thyroid cancer: a review of epidemiology, pathogenesis, and treatment. Journal of oncology.

[4] Kebebew E (2012). Anaplastic thyroid cancer: rare, fatal, and neglected. Surgery.

[5] Ricarte-Filho JC, Ryder M, Chitale DA, Rivera M, Heguy A, Ladanyi M, Janakiraman M, Solit D, Knauf JA, Tuttle RM, Ghossein RA, Fagin JA (2009). Mutational profile of advanced primary and metastatic radioactive iodine-refractory thyroid cancers reveals distinct pathogenetic roles for BRAF, PIK3CA, and AKT1. Cancer Res.

[6] Smallridge RC, Marlow LA, Copland JA (2009). Anaplastic thyroid cancer: molecular pathogenesis and emerging therapies. Endocrine-related cancer.

[7] Liu Z, Hou P, Ji M, Guan H, Studeman K, Jensen K, Vasko V, El-Naggar AK, Xing M (2008). Highly prevalent genetic alterations in receptor tyrosine kinases and phosphatidylinositol 3-kinase/akt and mitogen-activated protein kinase pathways in anaplastic and follicular thyroid cancers. J Clin Endocrinol Metab.

[8] Begum S, Rosenbaum E, Henrique R, Cohen Y, Sidransky D, Westra WH (2004). BRAF mutations in anaplastic thyroid carcinoma: implications for tumor origin, diagnosis and treatment. Modern pathology : an official journal of the United States and Canadian Academy of Pathology, Inc.

[9] Donghi R, Longoni A, Pilotti S, Michieli P, Della Porta G, Pierotti MA (1993). Gene p53 mutations are restricted to poorly differentiated and undifferentiated carcinomas of the thyroid gland. The Journal of clinical investigation.

[10] Garcia-Rostan G, Tallini G, Herrero A, D'Aquila TG, Carcangiu ML, Rimm DL (1999). Frequent mutation and nuclear localization of beta-catenin in anaplastic thyroid carcinoma. Cancer research.

[11] Smith N, Nucera C (2015). Personalized therapy in patients with anaplastic thyroid cancer: targeting genetic and epigenetic alterations. The Journal of clinical endocrinology and metabolism.

[12] Elliott DD, Sherman SI, Busaidy NL, Williams MD, Santarpia L, Clayman GL, El-Naggar AK (2008). Growth factor receptors expression in anaplastic thyroid carcinoma: potential markers for therapeutic stratification. Human pathology.

[13] Wiseman SM, Masoudi H, Niblock P, Turbin D, Rajput A, Hay J, Bugis S, Filipenko D, Huntsman D, Gilks B (2007). Anaplastic thyroid carcinoma: expression profile of targets for therapy offers new insights for disease treatment. Annals of surgical oncology.

[14] Ensinger C, Spizzo G, Moser P, Tschoerner I, Prommegger R, Gabriel M, Mikuz G, Schmid KW (2004). Epidermal growth factor receptor as a novel therapeutic target in anaplastic thyroid carcinomas. Annals of the New York Academy of Sciences.

[15] Schiff BA, McMurphy AB, Jasser SA, Younes MN, Doan D, Yigitbasi OG, Kim S, Zhou G, Mandal M, Bekele BN, Holsinger FC, Sherman SI, Yeung SC, El-Naggar AK, Myers JN (2004). Epidermal growth factor receptor (EGFR) is overexpressed in anaplastic thyroid cancer, and the EGFR inhibitor gefitinib inhibits the growth of anaplastic thyroid cancer. Clinical cancer research.

[16] Borbone E, Berlingieri MT, De Bellis F, Nebbioso A, Chiappetta G, Mai A, Altucci L, Fusco A (2010). Histone deacetylase inhibitors induce thyroid cancer-specific apoptosis through proteasome-dependent inhibition of TRAIL degradation. Oncogene.

[17] Ito Y, Yoshida H, Uruno T, Nakano K, Miya A, Kobayashi K, Yokozawa T, Matsuzuka F, Matsuura N, Kakudo K, Kuma K, Miyauchi A (2003). Survivin expression is significantly linked to the dedifferentiation of thyroid carcinoma. Oncology reports.

[18] Pannone G, Santoro A, Pasquali D, Zamparese R, Mattoni M, Russo G, Landriscina M, Piscazzi A, Toti P, Cignarelli M, Lo Muzio L, Bufo P (2014). The role of survivin in thyroid tumors: differences of expression in well-differentiated, non-well-differentiated, and anaplastic thyroid cancers. Thyroid : official journal of the American Thyroid Association.

[19] Isham CR, Bossou AR, Negron V, Fisher KE, Kumar R, Marlow L, Lingle WL, Smallridge RC, Sherman EJ, Suman VJ, Copland JA, Bible KC (2013). Pazopanib enhances paclitaxel-induced mitotic catastrophe in anaplastic thyroid cancer. Science translational medicine.

[20] Baldini E, D'Armiento M, Ulisse S (2014). A new aurora in anaplastic thyroid cancer therapy. International journal of endocrinology.

[21] Santarpia L, El-Naggar AK, Cote GJ, Myers JN, Sherman SI (2008). Phosphatidylinositol 3-kinase/akt and ras/raf-mitogen-activated protein kinase pathway mutations in anaplastic thyroid cancer. The Journal of clinical endocrinology and metabolism.

[22] Marlow LA, von Roemeling CA, Cooper SJ, Zhang Y, Rohl SD, Arora S, Gonzales IM, Azorsa DO, Reddi HV, Tun HW, Doppler HR, Storz P, Smallridge RC, Copland JA (2012). Foxo3a drives proliferation in anaplastic thyroid carcinoma through transcriptional regulation of cyclin A1: a paradigm shift that impacts current therapeutic strategies. J Cell Sci.

[23] Zhang L, Zhang Y, Mehta A, Boufraqech M, Davis S, Wang J, Tian Z, Yu Z, Boxer MB, Kiefer JA, Copland JA, Smallridge RC, Li Z, Shen M, Kebebew E (2015). Dual inhibition of HDAC and EGFR signaling with CUDC-101 induces potent suppression of tumor growth and metastasis in anaplastic thyroid cancer. Oncotarget.

[24] Mehta A, Zhang L, Boufraqech M, Zhang Y, Patel D, Shen M, Kebebew E (2015). Carfilzomib is an effective anticancer agent in anaplastic thyroid cancer. Endocrine-related cancer.

[25] Sadowski SM, Boufraqech M, Zhang L, Mehta A, Kapur P, Zhang Y, Li Z, Shen M, Kebebew E (2015). Torin2 targets dysregulated pathways in anaplastic thyroid cancer and inhibits tumor growth and metastasis. Oncotarget.

[26] Mehta A, Zhang L, Boufraqech M, Liu-Chittenden Y, Zhang Y, Patel D, Davis S, Rosenberg A, Ylaya K, Aufforth R, Li Z, Shen M, Kebebew E (2015). Inhibition of Survivin with YM155 Induces Durable Tumor Response in Anaplastic Thyroid Cancer. Clinical cancer research.

[27] Abaza MS, Bahman AM, Al-Attiyah R (2014). Superior antimitogenic and chemosensitization activities of the combination treatment of the histone deacetylase inhibitor apicidin and proteasome inhibitors on human colorectal cancer cells. International journal of oncology.

[28] Marlow LA, D'Innocenzi J, Zhang Y, Rohl SD, Cooper SJ, Sebo T, Grant C, McIver B, Kasperbauer JL, Wadsworth JT, Casler JD, Kennedy PW, Highsmith WE, Clark O, Milosevic D, Netzel B (2010). Detailed molecular fingerprinting of four new anaplastic thyroid carcinoma cell lines and their use for verification of RhoB as a molecular therapeutic target. The Journal of clinical endocrinology and metabolism.

[29] Chou TC (2010). Drug combination studies and their synergy quantification using the Chou-Talalay method. Cancer research.

[30] Wagle N, Grabiner BC, Van Allen EM, Amin-Mansour A, Taylor-Weiner A, Rosenberg M, Gray N, Barletta JA, Guo Y, Swanson SJ, Ruan DT, Hanna GJ, Haddad RI, Getz G, Kwiatkowski DJ, Carter SL (2014). Response and acquired resistance to everolimus in anaplastic thyroid cancer. The New England journal of medicine.

[31] Shaik S, Nucera C, Inuzuka H, Gao D, Garnaas M, Frechette G, Harris L, Wan L, Fukushima H, Husain A, Nose V, Fadda G, Sadow PM, Goessling W, North T, Lawler J (2012). SCF(beta-TRCP) suppresses angiogenesis and thyroid cancer cell migration by promoting ubiquitination and destruction of VEGF receptor 2. J Exp Med.

[32] Voorhees PM, Dees EC, O‘Neil B, Orlowski RZ (2003). The proteasome as a target for cancer therapy. Clinical cancer research.

[33] Altmann A, Markert A, Askoxylakis V, Schoning T, Jesenofsky R, Eisenhut M, Haberkorn U (2012). Antitumor effects of proteasome inhibition in anaplastic thyroid carcinoma. Journal of nuclear medicine.

[34] Wunderlich A, Arndt T, Fischer M, Roth S, Ramaswamy A, Greene BH, Brendel C, Hinterseher U, Bartsch DK, Hoffmann S (2012). Targeting the proteasome as a promising therapeutic strategy in thyroid cancer. Journal of surgical oncology.

[35] Orlowski RZ, Kuhn DJ (2008). Proteasome inhibitors in cancer therapy: lessons from the first decade. Clinical cancer research.

[36] Demo SD, Kirk CJ, Aujay MA, Buchholz TJ, Dajee M, Ho MN, Jiang J, Laidig GJ, Lewis ER, Parlati F, Shenk KD, Smyth MS, Sun CM, Vallone MK, Woo TM, Molineaux CJ (2007). Antitumor activity of PR-171, a novel irreversible inhibitor of the proteasome. Cancer research.

[37] Lai CJ, Bao R, Tao X, Wang J, Atoyan R, Qu H, Wang DG, Yin L, Samson M, Forrester J, Zifcak B, Xu GX, DellaRocca S, Zhai HX, Cai X, Munger WE (2010). CUDC-101, a multitargeted inhibitor of histone deacetylase, epidermal growth factor receptor, and human epidermal growth factor receptor 2, exerts potent anticancer activity. Cancer research.

[38] Wang J, Pursell NW, Samson ME, Atoyan R, Ma AW, Selmi A, Xu W, Cai X, Voi M, Savagner P, Lai CJ (2013). Potential advantages of CUDC-101, a multitargeted HDAC, EGFR, and HER2 inhibitor, in treating drug resistance and preventing cancer cell migration and invasion. Molecular cancer therapeutics.

[39] Shimizu T, LoRusso PM, Papadopoulos KP, Patnaik A, Beeram M, Smith LS, Rasco DW, Mays TA, Chambers G, Ma A, Wang J, Laliberte R, Voi M, Tolcher AW (2014). Phase I first-in-human study of CUDC-101, a multitargeted inhibitor of HDACs, EGFR, and HER2 in patients with advanced solid tumors. Clinical cancer research.

[40] Bible KC, Suman VJ, Menefee ME, Smallridge RC, Molina JR, Maples WJ, Karlin NJ, Traynor AM, Kumar P, Goh BC, Lim WT, Bossou AR, Isham CR, Webster KP, Kukla AK, Bieber C (2012). A multiinstitutional phase 2 trial of pazopanib monotherapy in advanced anaplastic thyroid cancer. The Journal of clinical endocrinology and metabolism.

[41] Smallridge RC, Copland JA, Brose MS, Wadsworth JT, Houvras Y, Menefee ME, Bible KC, Shah MH, Gramza AW, Klopper JP, Marlow LA, Heckman MG, Von Roemeling R (2013). Efatutazone, an oral PPAR-gamma agonist, in combination with paclitaxel in anaplastic thyroid cancer: results of a multicenter phase 1 trial. The Journal of clinical endocrinology and metabolism.

[42] Sosa JA, Elisei R, Jarzab B, Balkissoon J, Lu SP, Bal C, Marur S, Gramza A, Yosef RB, Gitlitz B, Haugen BR, Ondrey F, Lu C, Karandikar SM, Khuri F, Licitra L (2014). Randomized safety and efficacy study of fosbretabulin with paclitaxel/carboplatin against anaplastic thyroid carcinoma. Thyroid : official journal of the American Thyroid Association.

[43] Crawford LJ, Chan ET, Aujay M, Holyoake TL, Melo JV, Jorgensen HG, Suresh S, Walker B, Irvine AE (2014). Synergistic effects of proteasome inhibitor carfilzomib in combination with tyrosine kinase inhibitors in imatinib-sensitive and -resistant chronic myeloid leukemia models. Oncogenesis.

[44] Larrue C, Saland E, Boutzen H, Vergez F, David M, Joffre C, Hospital MA, Tamburini J, Delabesse E, Manenti S, Sarry JE, Recher C (2016). Proteasome inhibitors induce FLT3-ITD degradation through autophagy in AML cells. Blood.

[45] Hanke NT, Garland LL, Baker AF (2016). Carfilzomib combined with suberanilohydroxamic acid (SAHA) synergistically promotes endoplasmic reticulum stress in non-small cell lung cancer cell lines. Journal of cancer research and clinical oncology.

